# Compound Heterozygous Mutation of *Rag1* Leading to Omenn Syndrome

**DOI:** 10.1371/journal.pone.0121489

**Published:** 2015-04-07

**Authors:** Adam G. W. Matthews, Christine E. Briggs, Keiichi Yamanaka, Trudy N. Small, Jana L. Mooster, Francisco A. Bonilla, Marjorie A. Oettinger, Manish J. Butte

**Affiliations:** 1 Department of Molecular Biology, Massachusetts General Hospital and Department of Genetics, Harvard Medical School, Boston, MA 02114, United States of America; 2 Department of Biological Sciences, Wellesley College, 106 Central St., Wellesley, MA, 02481, United States of America; 3 Molecular Genetics Core Facility, Children’s Hospital Boston, Boston, MA 02115, United States of America; 4 Department of Dermatology, Mie University, Graduate School of Medicine, Mie 514–8507, Japan; 5 Bone Marrow Transplant Service, Memorial Sloan-Kettering Cancer Center, New York, NY 10065, United States of America; 6 Department of Pediatrics, Division of Immunology, Allergy, and Rheumatology, Boston Children’s Hospital, Boston, MA 02115, United States of America; 7 Department of Pediatrics, Division of Immunology, Allergy, and Rheumatology, Stanford University, 300 Pasteur Drive, Stanford, CA 94305, United States of America; Chang Gung University, TAIWAN

## Abstract

Omenn syndrome is a primary immunodeficiency disorder, featuring susceptibility to infections and autoreactive T cells and resulting from defective genomic rearrangement of genes for the T cell and B cell receptors. The most frequent etiologies are hypomorphic mutations in “non-core” regions of the *Rag1* or *Rag2* genes, the protein products of which are critical members of the cellular apparatus for V(D)J recombination. In this report, we describe an infant with Omenn syndrome with a previously unreported termination mutation (p.R142*) in *Rag1* on one allele and a partially characterized substitution mutation (p.V779M) in a “core” region of the other *Rag1* allele. Using a cellular recombination assay, we found that while the p.R142* mutation completely abolished V(D)J recombination activity, the p.V779M mutation conferred a severe, but not total, loss of V(D)J recombination activity. The recombination defect of the V779 mutant was not due to overall misfolding of Rag1, however, as this mutant supported wild-type levels of V(D)J cleavage. These findings provide insight into the role of this poorly understood region of Rag1 and support the role of Rag1 in a post-cleavage stage of recombination.

## Introduction

During the development of B cells and T cells, a diverse repertoire of antigen receptor genes are assembled from multiple component gene segments in a process known as V(D)J recombination [1]. Each of these gene segments is marked by a recombination signal sequence (RSS) that is recognized by a complex of the lymphoid-specific proteins Rag1 and Rag2. Together, Rag1 and Rag2 initiate V(D)J recombination by cleaving DNA to generate double-strand breaks consisting of two hairpinned coding ends and two blunt signal ends [2]. The ubiquitously expressed non-homologous end joining (NHEJ) proteins then collaborate with the Rag proteins to rejoin the cleaved DNA molecules, generating precise signal joints and imprecise coding joints [3].

Since antigen receptor gene assembly is required for the proper development of B cells and T cells, mutations that disrupt V(D)J recombination can lead to impaired immune function. Omenn Syndrome (OMIM 603554) is an autosomal recessive variant of severe combined immunodeficiency (SCID) with distinctive clinical features of generalized erythodermia, hepatosplenomegaly, and lymphadenopathy [4]. All patients with SCID are susceptible to infections from common bacteria and viruses as well as opportunistic and fungal pathogens. Unlike patients with classical SCID, patients with Omenn Syndrome have circulating T cells with an abnormal phenotype: they are typically poorly reactive, oligoclonal, and display cell-surface markers of previous activation [5]. B cells are typically absent or low and IgG levels are generally low while IgE levels are high.

Omenn Syndrome can be caused by mutations in *Rag1* and *Rag2* [6], or rarely by mutations in the NHEJ factor Artemis [7], in the IL-7 receptor alpha chain [8] or in the RNase mitochondrial RNA processing (RMRP) gene [9]. For many patients with Omenn Syndrome, the genetic defect remains unidentified [10]. In general, hypomorphic *Rag* mutations produce Omenn Syndrome, while null mutations produce T–B—SCID [11,12]. Interestingly, siblings with identical *Rag* mutations have developed either SCID or Omenn syndrome, suggesting that genetic or environmental effects can modify the phenotype of these disorders [12,13,14]. It has been suggested that early infections may be one factor that leads to the expansion of poorly reactive, oligoclonal T cells and the subsequent development of Omenn Syndrome instead of SCID [15]. Finally, hypomorphic *Rag* mutations can also cause a distinct SCID phenotype with an expanded pool of γδ T cells [16], combined immunodeficiency with granulomatous disease with or without autoimmunity [17,18], or autoimmune disease of varying severity [19,20,21].

Here, we report a boy with Omenn syndrome diagnosed at ~17 weeks of age. We discovered a maternally-inherited nonsense mutation on one allele of the *Rag1* gene. A missense mutation was identified on the other allele in a poorly characterized region of the Rag1 protein. To evaluate this mutation, we performed cellular V(D)J recombination assays, revealing that the maternal nonsense mutation is null, and the paternal missense mutation is severely hypomorphic. However, biochemical assays demonstrate that the paternal missense mutation does not affect catalysis of V(D)J cleavage *in vitro*, supporting that hypomorphic Rag1 activity in the post-cleavage stages of recombination is sufficient to confer susceptibility to Omenn Syndrome.

## Materials and Methods

### Clinical laboratory tests

Immunoglobulin levels and PRP and tetanus titers were determined by nephelometry at the Boston Children’s Hospital Chemistry Lab. Pneumococcal titers were determined by the Luminex method at ARUP Reference Laboratory. T cell subsets were determined in the Boston Children’s Hospital Immunology Lab by flow cytometry. T cell proliferations were determined at the University of Iowa using [^3^H]-thymidine incorporation during the last 24 hours of a 5-day culture. The control numbers for proliferations refer to an internal laboratory control; shipping controls were comparable. This study was approved by the Institutional Review Board at Boston Children’s Hospital. Both parents gave written informed consent.

### TCR spectratype assay

TCR spectratype (TCR-CDR3 length analysis) was performed with 24 TCR Vβ subfamily specific primers as well as CB primers as described previously[22]. Scoring of CDR3 profiles was performed by determining the number of contracted Vβ CDR3 size profiles in each subject's T cell CDR3 repertoire. Contracted profiles were defined as follows: oligoclonal (2–4 peaks), monoclonal (1 peak), or absent (no peaks detectable).

### DNA sequencing

Gene sequencing was performed using standard techniques (GeneDx, Inc.). Sequences were analyzed using Sequencher software (Gene Codes Corporation). The reference DNA and protein sequences for *Rag1* are from NIH RefSeq NM_000448.2 and NP_000439.1, respectively, and *Rag2* from NM_000536.3 and NP_000527.2, respectively. Nucleotide numbering starts with 1 at the A of the ATG translation initiation codon.

Other sequencing results were identified in this patient: A homozygous single nucleotide polymorphism (SNP) was identified in *Rag1*, c.2459A>G which codes for p.Lys820Arg (NIH RefSNP accession rs2227973). Two homozygous, non-synonymous, coding-region SNPs were found on *Rag2*, p.Val154Ala (RefSNP accession rs17852002) and p.Met322Thr (RefSNP accession rs17856658).

### Antibodies and plasmids

Antibodies used in this study were: anti-HA (clone HA-7, Sigma); anti-FLAG (clone M2, Sigma); anti-alpha Tubulin (clone DM1A, Abcam); and sheep anti-mouse IgG HRP-linked whole antibody (GE Healthcare). pGG49, pGG51, p3xFLAG-Rag2, and pcDNA6-myc-hisA-Rag1 were described previously [23]. Point mutations were generated by site-directed mutagenesis using Pfu Turbo DNA polymerase (Stratagene).

### Extrachromosomal V(D)J recombination assays

Extrachromosomal V(D)J recombination assays were performed in Br3neo human fibroblastoid cells as described previously [24], using either pGG49 (signal-joint formation) or pGG51 (coding-joint formation) as the reporter [25]. Briefly, we transfected human fibroblast cells with murine full-length Rag1, murine full-length Rag2, and a plasmid reporter substrate and harvested the cells at 48-hours post-transfection. The plasmid DNA encodes the gene for ampicillin (Amp) resistance, and further encodes a chloramphenicol (Cam) resistance gene preceded by a transcriptional terminator flanked by recombination signal sequences. Proper recombination confers Cam resistance. The plasmid DNA was isolated from the harvested cells and transformed into bacteria. V(D)J recombination frequency was measured by comparing the number of Amp^R^/Cam^R^ colonies to the number of Amp^R^ colonies [26]. Full-length Rag2 was transiently expressed using the p3xFLAG-CMV vector (Sigma). Wildtype Rag1, Rag1_V779M_, and Rag1_R142*_ were transiently expressed using the pcDNA6-myc-hisA vector (Invitrogen). Expression of all proteins was confirmed by Western analysis.

### Proteins

Recombinant FLAG-tagged full-length Rag2 was expressed by transient transfection of 293T cells and purified as described previously [27]. Recombinant wild-type core Rag1 (aa 387–1011) and Rag1_V779M_ were purified from *Escherichia coli* as described previously [28].

### In vitro V(D)J cleavage assays

Rag1 and Rag1_V779M_ were used for *in vitro* V(D)J cleavage assays as described previously [29]. Briefly, Rag1 (80 ng) and Rag2 (10 ng) were added to a 10 μL reaction mixture containing 25 mM HEPES (pH 7.5), 2 mM dithiothreitol, 60 mM potassium glutamate, 1 mM MnCl_2_, and 0.25 pmol of ^32^P-labeled DNA substrate. Reaction mixtures were incubated at 30°C for 2 hours, stopped with 95% formamide loading dye, separated by super-denaturing gel electrophoresis, and visualized by autoradiography.

## Results

### Clinical characteristics and laboratory findings

**Fig 1:**
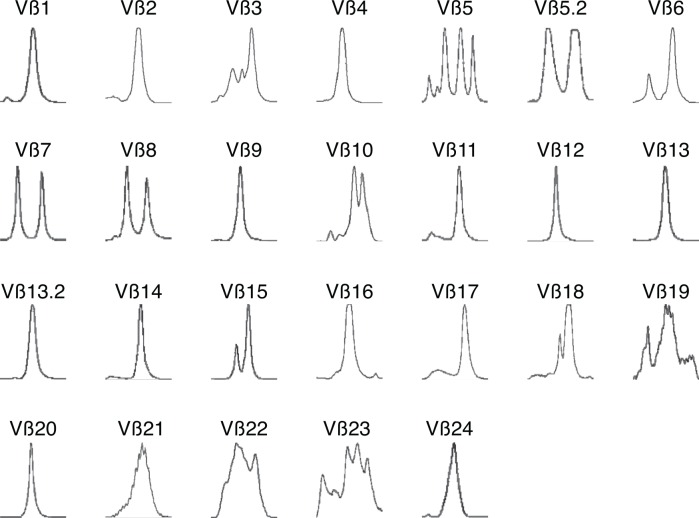
Collapsed T cell repertoire in Omenn Syndrome Patient. TCR Vβ spectratype analysis of CDR3 reveals profound oligoclonality and monoclonality, consistent with Omenn Syndrome.

P1 was born at term to non-consanguineous parents. Starting at one month of age, he developed recurrent, waxing and waning, scaling erythroderma with recurrent skin infections requiring oral antibiotics. At approximately 4 months, due to progression of his rash, he was referred to a dermatologist and underwent a skin biopsy, which showed acute micro-vesiculating, spongiotic dermatitis, dermal inflammation, and eosinophils. At 4.5 months of age, physical examination revealed global erythroderma, sparse, friable hair, generalized lymphadenopathy, and hepatosplenomegaly. Comèl-Netherton syndrome was ruled out by microscopic examination of scalp and eyelash hairs. Laboratory evaluation revealed hypoalbuminemia, peripheral eosinophilia, profound hypogammaglobulinemia, elevated IgE, absent isohemaglutinnins, and lack of specific antibody production (Table 1). Lymphocyte subset analysis showed 73% CD3+ T cells (absolute 3,015/μL), absent B cells, and 27% NK cells. The number of CD8+ T cells was mildly decreased and his CD4/CD8 ratio was elevated (Table 1). T cell proliferative responses to mitogens were severely depressed (Table 1), indicating a SCID-like disorder. Maternal engraftment was ruled out by analysis of micro-satellite markers by PCR (data not shown). A clinical diagnosis of Omenn Syndrome was made, supported by the finding of oligoclonal/monoclonal TCR beta chains by spectratype analysis (Fig 1). In addition, his CD4+ T cells showed an activated, memory phenotype, expressing CD45-RO and HLA-DR on 96% and 94% of cells, respectively (Table 1).

**Table 1:** Peripheral blood analysis of P1 at time of initial presentation (age 4½ months) is consistent with Omenn’s Syndrome.

	**Patient**	**Normal**
**Blood counts**		
White blood cell count (cells/μL)	16,270	7,000–16,000
Absolute lymphocyte count (cells/μL)	4,130	3,900–9,000
Absolute eosinophil count (cells/μL)	5,710	0–400
**Lymphocyte populations % (cells/**μ**L)**		(10^th^ to 90^th^ percentile)
CD3	73% (3,015)	51–77% (2,500–5,600)
CD4 (CD3 pos)	67% (2,767)	35–56% (1,800–4,000)
CD8 (CD3 pos)	8% (330)	12–23% (5,90–1,600)
CD19	0% (0)	5–10%
CD16 (CD3 neg)	27% (1,115)	5–15%
CD3 HLA-DR+	94%	2–10%
CD3 CD45-RO	96%	3–16%
**Immunoglobulins**		
IgG (mg/dL)	137	200–1200
IgA (mg/dL)	<7	5–46
IgM (mg/dL)	52	10–90
IgE (IU/mL)	5,550	10–30
**Lymphocyte proliferation**		
Background (cpm ± std)	134 +/- 37	1,012 +/- 566
ConA (cpm ± std)	2,133 +/- 600	86,229 +/- 16,286
PHA (cpm ± std)	4,140 +/- 532	83,383 +/- 6,314
PWM (cpm ± std)	10,100 +/- 1,560	63,575 +/- 4,996
Background (cpm ± std)	94 +/- 25	495 +/- 180
Tetanus (cpm ± std)	445 +/- 30	14,213 +/- 3,528
Candida (cpm ± std)	309 +/- 214	24,937 +/- 5,727

Normal values from the Children’s Hospital Laboratory, the Cincinnati Children’s Hospital Laboratory, or from reference [30].

At age 6.3 months, this patient underwent a T cell-depleted, HLA single-antigen mismatched, paternal peripheral blood transplant at Memorial Sloan-Kettering Cancer Center. Chemotherapeutic cytoreduction was achieved with intravenous busulfan (16 mg/kg) and cyclophosphamide (50 mg/kg). He received two doses of alemtuzumab (10 mg/m^2^/dose) prior to transplant for prophylaxis against graft rejection. Stem cells were depleted of T cells by CD34-positive selection followed by rosetting with sheep red blood cells. He received a total of 30.4 x 10^6^ CD34 cells/kg and 3.2 x 10^3^ CD3 cells/kg. Early post-transplant complications consisted of mucositis and persistent skin rash, which resolved by one month following HSCT. At approximately one-year after HSCT, he developed isolated severe myositis, presumed to reflect chronic GVHD, which required prolonged mechanical ventilation and treatment with steroids and cyclosporine A. He ultimately recovered, was able to breathe on his own, and was weaned off mechanical ventilation and immunosuppressive therapy. He has had no recurrence of his myositis. He was clinically well at age 5 years, when he was lost to follow-up. He has normal *in vitro* T cell function and specific antibody production following vaccination for Tetanus, *Haemophilus influenzae*, Pneumococcus, and polio virus.

### Termination mutation in Rag1 (p.R142*)

**Fig 2:**
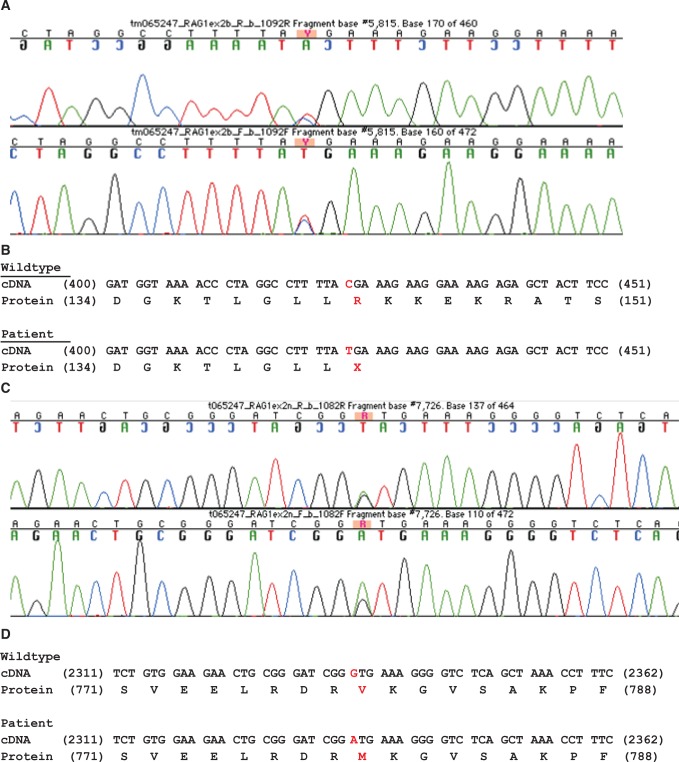
p.R142* maternal and p.V779M paternal mutations. P1 harbors a maternally inherited c.424C>T mutation, resulting in a premature stop codon. **A.** Sequencing chromatogram demonstrating the presence of a heterozygous c.424C>T mutation. **B.** Alignment of the wildtype and mutant Rag1 cDNA and protein sequences. c.424C>T creates a premature stop codon at position 142 of the protein. **C.** P1 harbors a paternally inherited c.2335G>A mutation, resulting in the non-synonymous coding mutation p.V779M. Sequencing chromatogram demonstrating the presence of a heterozygous c.2335G>A mutation. **D.** Alignment of the wildtype and mutant Rag1 cDNA and protein sequences. c.2335G>A creates a missense p.V779M mutation in the Rag1 protein.

On the basis of the patient history, physical exam, and laboratory findings, we diagnosed P1 with Omenn Syndrome. To determine the molecular etiology, we sequenced the *Rag1* and *Rag2* genes. In addition to previously characterized polymorphisms (see Methods), P1 had a heterozygous nonsense mutation in *Rag1* (c. 424C>T) (Fig 2A) resulting in an abnormal stop codon at p.Arg142Ter (Fig 2B). Sequencing of the mother’s genomic DNA revealed that this mutation was maternally inherited (data not shown).

Arg 142 is located within basic region I (aa 142–147) in the N-terminal “non-core” portion of Rag1. Assuming canonical start codon usage [31], truncation of the mRNA at this position would either result in nonsense-mediated decay of the transcript, or else could result in loss of >86% of the protein, including the core portion of the protein (aa 384–1008) that is essential for V(D)J recombination. Therefore, this nonsense mutation likely creates a null maternal allele.

### Missense mutation in Rag1 (p.V779M)

In addition to the maternal nonsense mutation, P1 also harbored a heterozygous missense mutation in Rag1 (c.2335G>A) on the paternal allele (Fig 2C), resulting in the non-conserved amino acid substitution of p.Val779Met (Fig 2D). Val 779 is located within the core portion of Rag1, between zinc finger B (aa 728–753) and basic region IV (aa 829–843). Since Val 779 does not fall within any known Rag1 functional motifs, it was unclear whether this mutation would affect the function of the Rag1/2 complex.

### Rag1_R142*_ is a null mutant and Rag1_V779M_ is a hypomorphic mutant

**Fig 3:**
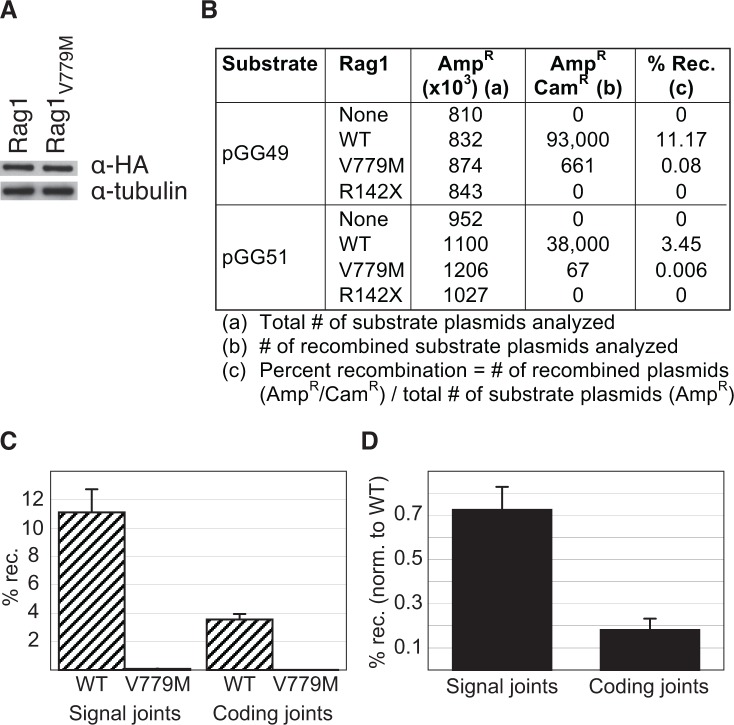
Rag1_R142*_ is a null mutant and Rag1_V779M_ is a hypomorphic mutant. A. Western analysis of Flag-tagged full-length Rag1 proteins expressed in Br3neo human fibroblast cells confirms that the wild-type (Rag1) and mutant (Rag1_V779M_) proteins are expressed at comparable levels *in vivo*. B. Representative recombination data from using the indicated constructs for transient V(D)J recombination assays in Br3neo cells. C. Absolute recombination activity using wild-type Rag1 (hatched) or the p.V779M mutant (shaded) with signal-joint substrates (left) or coding-joint substrates (right). Results represent the mean ±s.d. of six independent experiments. D. Normalized recombination activity of the p.V779M mutant. Recombination activity of the p.V779M mutant on each substrate was normalized to the activity of wild-type Rag1. Results represent the mean ± s.d. of six independent experiments.

To test whether the p.R142* and p.V779M mutations impairs the function of Rag1, we examined the ability of Rag1_R142*_ and Rag1_V779M_ to perform V(D)J recombination in cultured human cells using an extrachromosomal V(D)J recombination assay[24]. Fibroblast cell lines were then transfected with an expression vector encoding for either full-length wild-type Rag1, Rag1_V779M_, or Rag1_R142*_, plus both full-length Rag2 and an exogenous recombination substrate to detect either signal joints (pGG49) or coding joints (pGG51) [29]. Despite being expressed at comparable levels to wild-type Rag1 (Fig 3A), Rag1_V779M_ exhibited a dramatic deficiency (>99%) in V(D)J recombination activity (Fig 3B and 3C). Nonetheless, some residual activity was observed. The deficiency was observed on both types of substrates tested: those that form signal joints (0.73% of wild-type) and those that form coding joints (0.18% of wild-type) (Fig 3B and 3D). Although it has been reported that premature stop codons in the N-terminal region of Rag1 can be rescued by internal methionine usage [31], we observed no V(D)J recombination activity for Rag1_R142*_ (Fig 3B), and we were unable to detect expression of the Rag1_R142*_ protein. Therefore, the p.R142* mutation observed in P1 creates a null allele, while the p.V779M substitution observed in P1 is a hypomorphic mutation that profoundly impairs Rag1’s ability to catalyze V(D)J recombination.

### Rag1_V779M_ is functional in V(D)J cleavage assays in vitro

**Fig 4:**
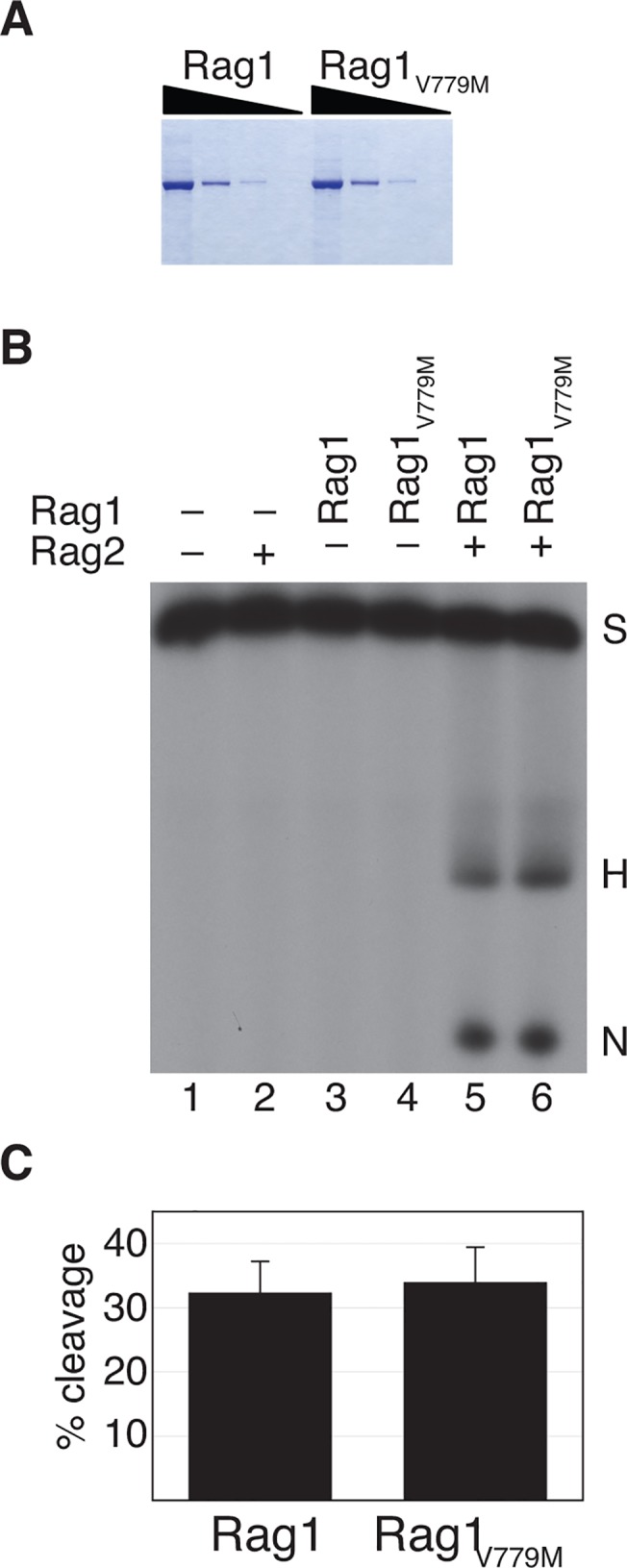
Rag1_V779M_ has wild-type V(D)J cleavage activity. **A**, Wild-type and mutant Rag1 proteins express and purify equally well. Coomassie stained gel of wild-type (Rag1) and mutant (Rag1_V779M_) recombinant core Rag1 proteins purified from E. coli. A serial dilution series (5-fold dilutions between lanes) is shown for each protein. **B**, The p.V779M mutant protein catalyzes wild-type V(D)J cleavage *in vitro*. Cleavage reactions were performed with recombinant wild-type core Rag1 and core Rag1_V779M_ in the presence of recombinant full-length Rag2 and resolved by denaturing polyacrylamide gel electrophoresis. The positions of the substrate (S) and cleavage products (hairpin (H) and nick (N)) are indicated. **C**, Absolute V(D)J cleavage activity of wild-type Rag1 (left) and the p.V779M mutant (right). Results represent the mean ± s.d. of four independent experiments.

Having observed that Rag1_V779M_ is deficient in V(D)J recombination activity, we wanted to test whether the p.V779M mutation impairs V(D)J cleavage. To generate a DNA double-strand break at a recombination signal sequence, the Rag1/2 complex must bind to the RSS and then catalyze both nicking and hairpinning. To assay for a defect in one of these steps of V(D)J recombination, we tested the ability of recombinant wild-type and mutant core Rag1 proteins (aa 387–1011) to catalyze V(D)J cleavage *in vitro*. The recombinant proteins, which expressed and purified equally well (Fig 4A), were incubated with full-length recombinant Rag2 plus a ^32^P-labeled DNA substrate that contains a recombination signal sequence. Surprisingly, Rag1_V779M_ catalyzed V(D)J cleavage at wild-type levels, generating both nicked and hairpinned products (Fig 4B and 4C). Thus, the p.V779M mutation does not impair Rag1/2-mediated RSS binding or cleavage.

## Discussion

We present here an infant boy with Omenn Syndrome bearing compound heterozygous mutations of *Rag1*: a nonsense mutation on the maternal allele (p.R142*) and a missense mutation on the paternal allele (p.V779M). Functional analysis of these *Rag1* alleles revealed that the p.R142* mutation creates a null allele, while the p.V779M substitution is a hypomorphic mutation that severely impairs V(D)J recombination *in vivo*. However, biochemical analysis demonstrated that the p.V779M substitution does not impair V(D)J cleavage *in vitro*, indicating that this mutation is not a catalytic mutation.

### Mechanism of Omenn Syndrome

Over the past 15 years, many different Rag mutations have been shown to cause Omenn Syndrome [6,13,32,33,34]. Indeed, more than 90% of Omenn Syndrome cases are attributable to hypomorphic mutations in Rag1 and Rag2 [35]. These hypomorphic Rag mutants can be broadly classified into two categories: those that exhibit impaired V(D)J cleavage *in vitro*—for example, mutants that are impaired in Rag1-Rag2 complex formation [11,36,37], DNA binding[11], or RSS nicking [38]; those that exhibit wild-type V(D)J cleavage activity *in vitro* but impaired V(D)J recombination *in vivo*—for example, mutants that are impaired in chromatin-binding [23,39] or in rejoining the broken DNA ends generated during V(D)J cleavage [40,41,42,43].

P1’s nonsense mutation on the maternal allele (p.R142*) would, if translated, remove all of the functional regions in Rag1 described above. As such, it either produces a severely truncated, nonfunctional protein or the message is deleted by nonsense-mediated decay. Therefore, the p.R142* mutation clearly falls in the first category of mutations that impair both V(D)J cleavage *in vitro* and V(D)J recombination *in vivo*.

**Fig 5:**
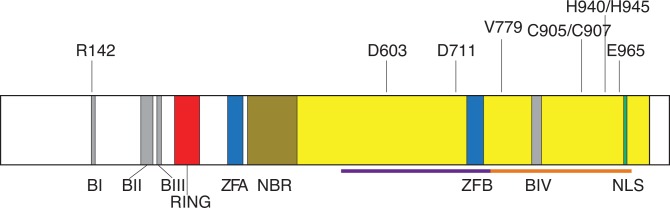
Overall domain structure of the human RAG1 protein. The human RAG1 protein is 1043 amino acid long and consists of a core region (aa 387–1011; yellow) and non-core regions (aa 1–386 and 1012–1043; white). There are two potential domains within the core region of Rag1: the central domain (aa 531–763; purple bar); and the C-terminal domain (aa 764–983; orange bar). Rag1 contains four basic regions (BI: aa 142–147; BII: aa 219–237; BIII: aa 244–252; BIV: aa 829–843; gray), a RING finger (aa 293–331; red), two zinc fingers (ZFA: aa 356–379; ZFB: aa 728–753; blue), a nonamer DNA-binding region (NBR: 387–457; dark yellow), a nuclear localization signal (NLS: aa 972–976; green), an Asp-Asp-Glu active site motif (D603, D711, E965), and two C-terminal zinc-binding sites (C905/C907 and H940/H945). The four basic regions serve as binding sites for the nuclear transport proteins Srp1 and Rch1. The RING finger functions as an E3 ubiquitin ligase and, together with zinc finger A, mediates Rag1 multimerization. Zinc finger B is thought to function as a Rag2 binding site. The positions of R142 and V779 are indicated.

P1’s missense mutation on the paternal allele (p.V779M) is more interesting in that it appears to leave the previously described functional regions of Rag1 intact (Fig 5), and yet, it severely impairs V(D)J recombination (Fig 3). The simplest explanation for this phenotype would be that the p.V779M substitution disrupts V(D)J recombination by causing misfolding of the protein, similar to other Rag1 mutants [44]. However, our *in vitro* V(D)J cleavage assays demonstrate that Rag1_V779M_ is fully functional in V(D)J cleavage, arguing against any substantial defect in protein folding. While a previous study suggested that Rag1_V779M_ might be modestly impaired in 12-RSS nicking *in vitro* [38], we found no apparent defect in the catalytic activity of this mutant. Although it is difficult to account for differences in protein purifications and experimental assay conditions, based on the robust cleavage activity that we observed, we would argue that the p.V779M mutation likely falls in the second category of mutations that exhibit wild-type V(D)J cleavage activity *in vitro*, but impaired V(D)J recombination *in vivo*. It is possible that Rag1_V779M_ is deficient in V(D)J recombination because it fails to interact with some key regulatory factor *in vivo*, and is therefore deficient in coupled cleavage, analogous to the Rag2 mutations described in other Omenn Syndrome patients [23,45]. Alternatively, it is possible that Rag1_V779M_ impairs the rejoining of the broken DNA ends generated during V(D)J cleavage. Along these lines, it is interesting to note that Rag1_V779M_ is more severely impaired in coding joint formation than it is in signal joint formation (Fig 3D), suggesting that Rag1_V779M_ may be a joining-deficient mutant that affects the post-cleavage stage of V(D)J recombination, analogous to previously identified Rag mutants [40,41,42,43]. Future studies will test the hypothesis that hypofunctional Rag1 and Rag2 mutants can confer susceptibility to Omenn Syndrome by impairing the post-cleavage stage of V(D)J recombination.

### Function of the Rag1 C-terminal domain

Previous studies using limited proteolysis have identified two potential domains within the core portion of human Rag1: the central domain (aa 531–763); and the C-terminal domain (aa 764–983) [46]. There are four known functional elements within the Rag1 C-terminal domain: basic region IV (aa 829–843), which binds to the nuclear transport protein Rch1 [47]; the nuclear localization signal (aa 972–976) [48]; E965, which is one of three key active site residues [49,50]; and two zinc-binding sites (C905/C907 and H940/H945) [51]. Although P1’s missense mutation (p.V779M) lies within the Rag1 C-terminal domain, and dramatically affects V(D)J recombination *in vivo*, it does not affect any of the known functional elements in this region. Does V779 lie within a new functional region of the Rag1 C-terminal domain? A recent study took a systematic approach to correlate the recombination activity of mutant human Rag1 proteins to the clinical and immunological presentation of the patients harboring these mutations [52]. Intriguingly, this study found several other mutations flanking V779 that also dramatically impaired V(D)J recombination *in vivo*: p.R764P; p.Y768*, p.E770K, p.R778W, and p.R786L. In light of these findings, it is tempting to speculate that V779 lies within a functionally significant region of the Rag1 C-terminal domain, possibly facilitating the proper repair of signal ends and coding ends via the classical non-homologous end-joining pathway.
